# Translating the molecular hallmarks of colorectal cancer to patient therapies: an interview with Owen Sansom

**DOI:** 10.1242/dmm.017350

**Published:** 2014-08

## Abstract

Owen Sansom, Deputy Director of the Cancer Research UK Beatson Institute, began his research career investigating the molecular mechanisms of apoptosis. Over the course of his work he has moved progressively into a more translational arena, and the current focus of his lab is to understand the signalling pathways underlying colorectal and pancreatic cancers. The Sansom lab uses mouse models to pinpoint how mutations that commonly occur in these frequently deadly cancers co-operate to promote tumorigenesis *in vivo*. This work has provided many important insights into the molecular changes associated with intestinal and pancreatic neoplasia and has revealed new targets for drug development. Here, Owen tells the stories behind some of his most exciting breakthroughs, describes the experiences and mentors that shaped his research interests and style of running a lab, and discusses the challenges of recapitulating the complexity of cancer as well as translating preclinical evidence to patient therapies.

Owen Sansom was born in Eastbourne in 1975. He obtained his BSc degree in Genetics from the University of Nottingham, UK in 1996, followed by a Master’s degree in Biology from the University of Manchester, UK. He then moved to Edinburgh to investigate the role of DNA-damage-induced apoptosis in cancer, under the supervision of Alan Clarke and Andrew Wyllie. Continuing his post-doctoral training in the Clarke lab, Owen made the groundbreaking discovery of the *in vivo* role of the tumour suppressor protein APC and Wnt signalling in intestinal cancer, which formed the foundation of followup work performed in his own group at the Cancer Research UK (CRUK) Beatson Institute, Glasgow. Since setting up the lab in 2005, he has helped define the molecular hallmarks of colorectal cancer (CRC), as well as pancreatic cancer, and has used mouse models to identify potential therapeutic targets for these aggressive malignancies. Among his many contributions to the field of cancer biology, Owen and collaborators showed that stem cells can drive the rapid development of cancer in the intestinal epithelia, lending weight to the ‘cancer stem cell’ theory. In 2007, Owen received the BACR/AstraZeneca Young Scientist Frank Rose Award. He is now Deputy Director of the CRUK Beatson Institute and Fellow of the Royal Society of Edinburgh. In 2013, he was appointed as a Monitoring Editor of *Disease Models & Mechanisms* (DMM).

**Have you always been interested in science, and are you where you pictured you would be 10 years ago?**

I grew up on a farm and was always interested in nature and how things work. Growing up I felt I wanted to do something in science but wasn’t quite sure exactly which route I would follow. Academically, I was good at biology but also keen on history, so spent a bit of time deciding which course I was going to do at university. I opted for Genetics at the University of Nottingham because the course offered a good mix of classic molecular biology alongside theoretical evolutionary genetics. From there I just became more interested in the topic as I learned more about it. As I moved from theory to practical science I became more and more engaged.

**Figure:**
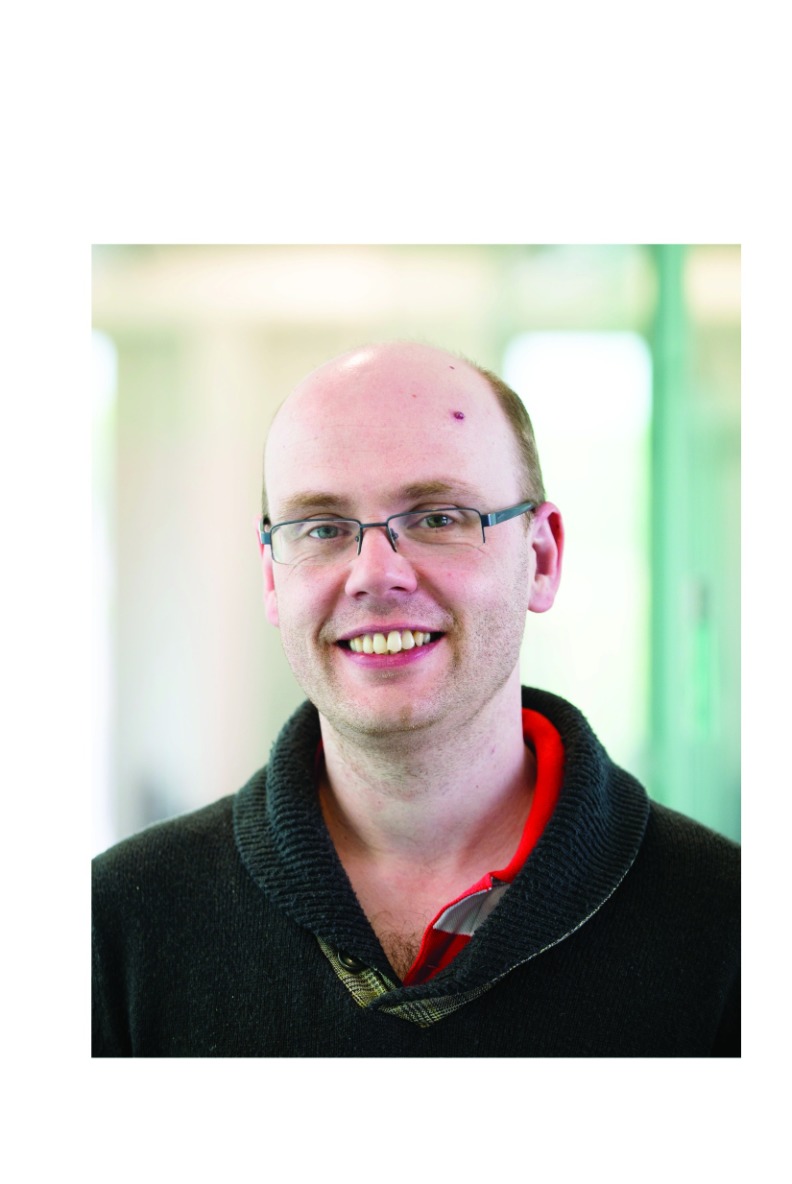


When you’re young you’re not 100% sure what you want to do, and I think a lot of people end up in science after gaining some initial experience and realising that they’re a good ‘fit’ for it. And as you switch from theoretical lecture-based science to a practical scenario you begin to realise whether you are able to do it or not. Often you get people who are very intelligent but when they go into a lab they’re a bit of a disaster. In science it can take years for things to come to fruition and provide answers, and getting used to failing is something you have to come to terms with really quickly. I’m pretty stoical when it comes to the pitfalls as well as the successes, and I feel very lucky to be where I am.

**How did your early training shape your current research interests?**

During my Master’s degree I worked in labs that were interested in apoptosis. At the time there was a lot of excitement surrounding this type of cell death, and I really wanted to learn more about the process and the signals involved. I ended up in Edinburgh for my PhD, working with Andrew Wyllie and Alan Clarke. Andrew is one of the people who, working as a pathologist at the University of Edinburgh, discovered apoptosis many years ago. Since that initial discovery, he’d done a lot of the molecular work behind several milestones, such as uncovering the role of p53 in apoptosis, and so it was very exciting to be working in his group. Alan was very much at the forefront of making genetic knockout mice and looking at how apoptosis affects carcinogenesis *in vivo*. So, whereas in Manchester [for my Master’s degree] I’d been doing molecular mechanistic studies, in Edinburgh I started thinking more about the broader picture – how important this process is in cancer and how we can target it. These are big questions that remain largely unanswered to this day, and my group is actively working on them.

“In science it can take years for things to come to fruition and provide answers, and getting used to failing is something you have to come to terms with really quickly”

**Was that your first foray into translational science?**

I think we almost fell into translational work, as our models became good mimics of human cancer and we were very keen to manipulate pathways *in vivo* using available drugs, to help advance our biological understanding of the disease. The translational side of things was, and still is, very interesting for me, and a lot of our work is focused on trying to understand fundamental biological processes and then how they go wrong in cancer. Cancer, as a complex system to study, is really quite fascinating, and studies using protein inhibitors, for example, can give insights into the disease but also the physiological mechanisms in normal cells. The discovery of drug compounds to use in this kind of study has helped basic biology immensely, as well as driving translation into the clinic. We’re interested in colon cancer, and our approach has been to generate mouse models with mutations in genes that ‘go wrong’ and use these models to work out what happens downstream of loss of *APC*, the tumour suppressor gene that is mutated in 80% of patients. These models really recapitulate the complexity of human cancer and so provide a good system for analysing the pathways involved. Going forward, these models will be important for translational studies and drug discovery.

**Taking a step back, could you explain why colon cancer is such an important clinical problem?**

There are a number of reasons. It is a very common cancer – there are literally thousands being diagnosed with the disease every year – and it’s the third most common cause of cancer-associated death. When the tumour is small, it is easy to surgically remove and most colon cancers can be cured by surgery. But, at a late stage, metastasis to the liver occurs and this is the cause of cancer mortality. The high incidence of the malignancy and its ability to spread is why it is such a challenging disease, although recently we have seen some major improvements in 5-year survival rates for those late stages, mostly attributable to surgery of metastatic disease.

**In 2009 you published a paper with Hans Clevers and Nick Barker describing the role of stem cells in the colon. What was the story behind that study?**

In 2002/2003, I was at a meeting focused on the phenotypes of *APC* loss and it was at the time that Hans Clevers’ lab was looking at the role of β-catenin in stem cells and the gut. Our phenotypes were very similar – when we deleted *APC*, what went up was similar to what went down when they knocked down β-catenin in colon cancer cell lines. We began chatting and comparing our list of targets, and started collaborating from there. I was always surprised that Hans wanted to collaborate with us because his lab was doing so well!

Around that time we started thinking about the origin of cancer – a very fundamental and important question. We wanted to work out whether different cells within the intestine have different capacities to be transformed. Hans and Nick had produced a very nice mouse model that allowed them to target Cre-mediated deletion of *APC* in Lgr5-marked stem cells. Together, we found that deletion of *APC* caused rapid development of adenomas from these stem cells, proving that a tumour could form very quickly from a stem cell route. It showed us that all you need is to lose *APC* within these cells to give you a benign tumour very rapidly. The flip side of that paper was the discovery that targeting *APC* deletion to non-stem-cell populations causes very few lesions. One of the nice things about the paper was this ‘compare and contrast’ result for stem-cell and non-stem-cell populations – by working with Hans and Nick, we were able to tackle the question from both sides.

**What are the main implications of the findings?**

If we think about the intestinal epithelium as a kind of escalator, where cells are born at the bottom, divide, differentiate and then fall off at the top, it is interesting that *APC* loss can reverse many of these properties and thus a single mutation can effectively act as a perfect storm for CRC initiation. Loss of a single tumour suppressor in a stem cell is sufficient for tumorigenesis.

One other thing to bear in mind, however, is that colon cancer can take many years to develop, so you can imagine that cancers that develop rapidly from stem cells might respond differently to treatments than cancers that develop via slightly longer routes and need other mutations to accumulate. Recently, in collaboration with Florian Greten in Munich, we have been trying to model the formation of tumours from differentiated populations of the intestine by adding additional mutations that are common in CRC (e.g. *KRAS* mutation) alongside *APC* mutations.

We know from studies showing that a fibroblast can be turned into an embryonic stem cell that the plasticity of cells is immense. Our data (published recently in *Cell*) show pretty convincingly that we can form tumours from dedifferentiated populations. The key now is to apply this to human CRC. It is quite possible that tumours arising from different cell types, for example differentiated versus stem cells, may have very different properties. Tumours emerging from differentiated cells would be exposed to different environments (such as lower levels of Wnt ligands, higher levels of TGFβ ligands, closer proximity to bowel microorganisms) that may select for different mutations to allow tumour progression.

**What are the main advantages of using mouse models to study the dynamic process of tumorigenesis?**

Genetically engineered mouse models allow us to introduce the same mutations that occur in humans into the mouse, and also into a small subset of cells. This means we can mimic the cancer process much better. We’re increasingly recognising the complexity of cancer; the idea that you need multiple mutations and that, when cancer therapies fail, resistance emerges, all within a complex tumour architecture influenced by the immune system and neighbouring cells. Genetically engineered mice give us the capacity to grow complex tumours that co-evolve a vasculature and stroma. The more we learn about cancer, the more importance we are placing on the immune system and stromal environment. The tumour stroma provides masses of signals and growth factors as well as support structures. Given all these signals flying around, targeting one pathway or one node is probably never going to be sufficient – there’s no ‘magic bullet’. We use mouse models to try to work out how different signalling pathways integrate to arrive at the cancer state, so we can take a multi-pronged approach to therapy. The development of 3D organoids by the Clevers group has revolutionised our *in vitro* work on the intestine. There is a great excitement that tumour organoids or spheroids will be excellent for predicting tumour response but we do have to ensure that we have the most appropriate stroma so that we can capture the complexity and difficulty of inhibiting endogenous tumours.

“We’re increasingly recognising the complexity of cancer; the idea that you need multiple mutations and that, when cancer therapies fail, resistance emerges, all within a complex tumour architecture influenced by the immune system and neighbouring cells”

Mouse models also give us the potential to be able to test the concept of stratified medicine. One of the biggest aims in clinical testing at the moment is targeting the therapy to the right patient. We envisage being able to put the major driver mutations into mice and saying ‘Ok, I’ve got a tumour that has a mutation in X and Y, does this really respond as we would predict in this setting?’. This would be preferable to working on a panel of established cell lines that might evolve to be very different in culture to a real human tumour. If you get a really striking result then you can be pretty sure that you can predict what is going to happen in human clinical trials. Showing what doesn’t work, i.e. lack of response, is just as important as showing what does work. With increasing demand for models that accurately recapitulate cancer and are predictive of drug response, journals such as DMM become more and more important.

**You’ve mentioned that modelling the complex environment of the tumour is one of the key challenges in understanding cancer biology. What would you say are the most urgent challenges in terms of translation and drug discovery?**

Trying to get clinicians and pharmaceutical companies to have enough confidence in preclinical observations to move from experimental systems into a clinical trial is still a big challenge. As we go forward I really hope that we will be using mice less and people more. I do quite a bit of work on pancreatic cancer, as well as on colon cancer. There have been recent advances in chemotherapy for pancreatic cancer, but the disease is still lethal. Most patients with non-surgically resectable tumours do not survive for more than 6 months. There are potential benefits of taking highly robust preclinical observations and moving them swiftly into patients in this scenario.

“Trying to get clinicians and pharmaceutical companies to have enough confidence in preclinical observations to move from experimental systems into a clinical trial is still a big challenge”

Our biggest challenge as a community is deciding what we think is good enough and robust enough evidence to take into patients. At what point can we say ‘yes, our scientific evidence is so strong we believe this should move forward to the clinic now’? How do we accelerate this process; how do we come up with a platform or a decision-making tree that helps us to know when to believe preclinical data? Related to this is the concept of the irreproducibility of science. How do we address this and convince the sceptics? I think that it’s critical to come together as a community, and set up networks where people are challenging each other sufficiently so that our data are proven to be robust and reproducible across a number of centres. This would also mean that we’re all testing our drugs in the same way and following the same procedures.

I think we always need to keep an eye on the big picture and remember why we’re doing what we’re doing. Recently, I had a look at which clinical trials are currently underway across the UK and felt that there were a number, particularly with an immunotherapy and immunological focus, where, as a scientist, you were scratching your head to understand the rationale. We have to move to a system where trials are initiated with the strongest possible preclinical data, and where it is easy for researchers with striking results from preclinical models to be able to influence clinical trial design. Currently, it appears that if a well-funded company wants to run a trial this is relatively straightforward even if there appears to be little rationale for it.

**From a personal standpoint, what would you say your most exciting research breakthrough has been?**

With science you have lots of highs and lows and you’re generally most excited about your most recent results. But, reporting the intestinal phenotype after *APC* gene deletion as a post-doc in Alan’s lab probably remains my most exciting result to date. We were the first group to delete *APC* in the intestine, and it was incredible to observe the robust, strong phenotype and then go on to characterise the pathways that go wrong. Within five days we had managed to completely rewire intestinal homeostasis. This work kind of founded my own lab as well as forming the basis of Alan’s lab for a long time.

Nowadays, we’re very excited about the mechanisms of cooperation and synergy between oncogenes and tumour suppressor genes. We have some very nice recent data (with Professor Anne Willis) showing that, following *APC* loss, it is important for the translational machinery to become more active, so that sufficient protein is produced to allow *APC*-deficient cells to retain their proliferative phenotype.

I also work in areas that I never thought I would end up focusing on, such as cancer metabolism. When you are taken outside your comfort zone and are finding out amazing things, it can be very exciting. Of course, these new questions come with a bit of worry about all the reading you need to do to get up to speed. But that’s the other exciting thing – being able to challenge yourself by moving into new areas and broadening your questions.

**You have collaborated quite a lot in your career. Are there any particular mentors who have influenced your style of doing research and running a lab?**

Alan Clarke is basically one of the most brilliant people I’ve ever met. He’s very intelligent, very laid back, good fun and I am inspired by his overall attitude, although I’m not as laid back as Alan. The set-up in his lab allows people to do amazing work whilst being in an environment where everyone is happy and works together. This is what you aspire to as a group leader – you want a lab where everyone is engaged, so that the ideas are fizzing off people. Alan has been a strong influence in my career.

I have also done a lot of work with Doug Winton in Cambridge over the years. Doug’s approach is to do beautiful science, no matter how long it is going to take. He has worked out so many important questions in terms of intestinal stem cells, the neutral drift hypothesis of stem cells and also stem cells within tumours. He has established himself as one of the leaders in the stem-cell field. I always look forward to reading his work, and as a person he is also really nice and down-to-earth. Since the initial *Nature* paper, I have also continued to work with Hans [Clevers] and Nick [Barker], which has been very valuable and exciting.

Finally, Margaret Frame [ex Deputy Director of CRUK Beatson Institute, now head of the CRUK Edinburgh Cancer Centre] and Karen Vousden [Director CRUK Beatson Institute] were very supportive when I set my laboratory up at the Beatson Institute. They really helped guide me through many of the pitfalls of setting up your laboratory. Also they set up an excellent collaborative atmosphere that really helped my group broaden its interests. For example, Margaret and I decided to move into pancreatic cancer after a long chat over coffee one day and, with the support of the institute, we were able to get this going relatively quickly.

**What advice do you pass on to young researchers hoping to follow in your footsteps?**

A scientific career is based on the research that you do and I think the important thing is to always keep the scientific question in mind and go where the data takes you. Don’t start with any preconceptions. No matter what happens, it is just very interesting to see how your story evolves.

At a time when we’re all very conscious of getting publications, I think it’s still important to build a career where you build an area of research. For example, my laboratory is still working on the *APC* gene and using similar models to over 10 years ago; there are still so many questions to answer and, as new technology develops, you can learn more.

The other thing is to do what interests you. You never know quite where your research will take you so you need to be aware of what is about. You need to read, you need to understand and then you need to be the one driving your project. You should know your project much better than anybody else.

“With the review process you have to become battle-weary, and remember that even at his best Tiger Woods lost more golf tournaments than he won – that’s publishing”

Finally, don’t get too disheartened when your papers are rejected. It is just one of those things – ignore it and move on. If there is a very good point that is raised then it is important to address and you gain from the experience. With the review process you have to become battle-weary, and remember that even at his best Tiger Woods lost more golf tournaments than he won – that’s publishing. Even at your peak you’re going to have more papers rejected than accepted, so you just have to take what’s useful from the review process, brush yourself off and submit it somewhere else.

**How do you relax and have fun away from the lab?**

Living in Scotland I very much enjoy going to the pubs and going for meals out. The scenery is wonderful, so I also like going for long walks. Although I get a bit tired of travelling because I do a lot for work, I do enjoy seeing new places and see the travel as something of a perk. So, a walk, a good pint of beer and then a holiday every now and again.

